# Enhanced Performance of Carbon Nanotube Immobilized Membrane for the Treatment of High Salinity Produced Water via Direct Contact Membrane Distillation

**DOI:** 10.3390/membranes10110325

**Published:** 2020-10-31

**Authors:** Madihah Saud Humoud, Sagar Roy, Somenath Mitra

**Affiliations:** Department of Chemistry and Environmental Science, New Jersey Institute of Technology, Newark, NJ 07102, USA; msh36@njit.edu (M.S.H.); sagar@njit.edu (S.R.)

**Keywords:** produced water, membrane fouling, membrane distillation, carbon nanotube, antiscalants, desalination

## Abstract

Membrane distillation (MD) is a promising desalination technology for the treatment of high salinity water. Here, we investigated the fouling characteristics of produced water obtained from hydraulic fracturing by implementing a carbon nanotube immobilized membrane (CNIM) via direct contact membrane distillation. The CNIM exhibited enhanced water vapor flux and antifouling characteristics compared to the pristine membrane. The normalized flux decline with the polytetrafluoroethylene (PTFE) membrane after 7 h of operation was found to be 18.2% more than the CNIM. The addition of 1-Hydroxy Ethylidene-1, 1-Diphosphonic acid (HEDP) antiscalant was found to be effective in reducing the membrane fouling. The salt deposition on the membrane surface was 77% less in the CNIM, which was further reduced with the addition of HEDP in the feed by up to 135.4% in comparison with the PTFE membrane. The presence of carbon nanotubes (CNTs) on the membrane surface also facilitated the regenerability of the membrane. The results indicated that the CNIM regained 90.9% of its initial water flux after washing, whereas the unmodified PTFE only regained 81.1% of its initial flux after five days of operation.

## 1. Introduction

Hydraulic fracturing or fracking employs horizontal drilling techniques to release oil and hydrocarbon by injecting high pressure water containing particulates and chemical additives. A major problem with fracking is the large volume of wastewater it generates, both initially as “frac” flow back, and over time as produced water. The U.S. alone generates approximately 25 billion barrels per year from oil and gas activities [1]. This amount is increasing as fracking activities expand. At the same time, options for waste water disposal are narrowing, putting oil and gas operations in jeopardy. Water pollutants in frack and produced water include dissolved solids, oil and grease, suspended solids, volatile organics, heavy metals, dissolved gases, chemical additives such as scale and corrosion inhibitors, guar gum and emulsion/reverse-emulsion breakers [2,3]. After the recovery of some of the additives, desalination and total dissolved solids (TDS) removal remain the major challenge, especially as the concentration can be as high as 350,000 mg/L, that is, ten times higher than sea water [4].

The two major approaches to desalination (or desalting) are thermal distillation and membrane separation. In the former, the saline water is boiled using thermal energy and re-condensed. Commercial thermal distillation methods such as multistage flash (MSF), multi-effect (ME) distillation and mechanical vapor compression (MVC) have relatively large footprints and require significantly higher investments. On the other hand, membrane processes have lower capital and operating costs and the most common desalination technique is reverse osmosis (RO) [5]. Some of the limitations of RO result from the increase in osmotic pressure at high salt concentrations, which often makes it ineffective for treating highly saline water (concentrations above 70,000 ppm). Moreover, the dense hydrophilic membranes used in RO tend to foul easily, which leads to low water production and reduced membrane life. Therefore, RO often requires extensive pretreatment such as water softening, which increases both capital and operational expenses. Therefore, there is an urgent need to develop new membrane-based techniques for treating oil and gas industry produced water [6,7]. 

At this point, the two most viable techniques for treating high salinity water appear to be forward osmosis (FO) [8,9] and membrane distillation (MD) [10,11]. Although, FO exhibits promising results, the regeneration of draw solution and recovery of pure water requires additional separation processes [12,13]. On the other hand, MD is a thermally driven desalination technology that has already shown steady improvement in the design of membranes and in its technical performance [14,15]. Previous studies have shown that MD has the potential to achieve a salt rejection rate of up to 99.9% [16,17] and to remove 99.5% of the organic materials [18]. Moreover, the low operating temperature (from 60 to 90 °C) of MD also makes it ideally suited for integration with renewable energy sources such as solar or low grade industrial waste heat sources [19,20] such as flare gas at oil fields [6].

Recently, polymeric membrane modification through the incorporation of nanomaterials (NMs) has led to improved water permeability and removal efficiency. These NM-enabled membranes are able to address some common limitations of conventional membranes, such as low flux and fouling [21,22]. Robust, free standing porous TiO_2_ nanowire membranes employed in water treatment applications have shown improved photocatalytic degradation of environmental pollutants [23] and Fe-based polymer nanocomposite membranes have been successfully fabricated via electrospinning to remove Cd^2+^ ions from water [24]. Carbon nanotube (CNT)-based composite membranes have shown exceptional separation performance due to the unique properties of CNTs, such as the high aspect ratio, atomic scale smoothness and chemical inertness and sorption properties that play critical role in the transport of water molecules [25]. Our group has fabricated carbon nanotube-based membranes using the simple approach of immobilizing the nanotubes on porous supports, and have reported their application in a variety of separations ranging from pervaporation, extraction to nanofiltration [26,27,28,29]. The physicochemical interaction between the water molecules and the membrane can be dramatically altered by immobilizing CNTs on the membrane surface [30,31,32]. First, CNTs are excellent sorbents that have surface areas between 100 and 1000 m^2^/g [33]. Many factors, such as the presence of defects, capillary forces in nanotubes, the polarizability of the honeycomb graphene structure lead to strong H_2_O vapor/CNT interactions; and the absence of a porous structure leads to high specific capacity while facilitating fast desorption of large molecules. It is anticipated that the carbon nanotube immobilized membrane (CNIM) will provide higher flux in the treatment of produced water [34,35]. A major obstacle in the widespread use of membrane technologies during the treatment of high salinity water is membrane fouling [36,37] due to the deposition of suspended or dissolved substances on the membrane surface and/or within its pores [38,39]. This is particularly true for produced water that contains high levels of salts, ions and metals. With a high concentration of CaCO_3_ and different cations at near saturation level, any membrane process including MD is expected to foul rapidly. Recently, we reported that CNIM shows relatively lower fouling as the CNTs act as nano-brushes that prevent the salt crystals from depositing on the surface [40]. The CNIM has shown high salt tolerance compared to pure polymeric membranes and has been used to treat water with TDS as high as 230,000 mg/L [41].

The use of antiscalant has also been reported to be beneficial in RO and other processes by reducing scaling of different salts [42,43]. Various types of antiscalants including acids, bases, enzymes, surfactants, disinfectants and combined cleaning materials has been employed in membrane separation processes [44,45]. The choice of antiscalants depends on the nature of treated water. During oil and gas extraction, a large volume of waste water referred to as “produced water” is generated as a byproduct of the drilling operation, which needs to be treated to minimize the environmental damage. The quality of the produced water varies depending upon the geographical location, and the total dissolved solids concentration can reach as high as 300,000 mg/L, which poses a significant challenge. Therefore, there is growing interest in possible options for treatment or reuse of this water. Produced water contains iron-based components that deposit on the membrane surface even with relatively low concentrations of iron in the feed side. 1-Hydroxy Ethylidene-1, 1-Diphosphonic acid (HEDP) is a threshold inhibitor based on phosphonic acids (or their salts), which have the added advantage of sequestering iron in a stoichiometric reaction [46,47,48]. This is important in membrane applications as any soluble iron will cause rapid fouling as it oxidizes and becomes insoluble. HEDP has the potential to dissolve the oxidized materials on the surface of these metal [49]. The objective of this paper was to investigate the enhancement in water vapor flux and the antifouling characteristics of CNIM in the treatment of high salinity, fouling-prone produced water via direct contact MD (DCMD). Yet another objective is to study the effect of antiscalant HEDP in reducing fouling.

## 2. Materials and Methods

### 2.1. Chemicals and Materials

Produced water used in this experiment was collected from Chemtreat Company (Philadelphia, PA, USA). Deionized water (Barnstead 5023, Dubuque, IA, USA) was used in all experiments. Filter papers (Whatman 1441-150, size 41 with a diameter of 150 mm) came from Cole-Parmer (Vernon Hills, IL, USA). Multi-walled CNTs (MWCNTs) were purchased from Cheap Tubes Inc., Brattleboro, VT, USA. The average diameters of the CNTs were ~30 nm and the length ranged up to 15 µm. 1-Hydroxy Ethylidene-1,1-Diphosphonic Acid (HEDP) antiscalant was purchased from Fisher Scientific Company (Hanover Park, IL, USA).

### 2.2. Water Sample Composition

The composition of the produced water samples used for the experiments is shown in Table 1. The pH and the conductivity of the sample used in this study was 2.19 and 239,651 µmho, respectively.

### 2.3. Water Sample and Pretreatment Methods

**Figure 1:**
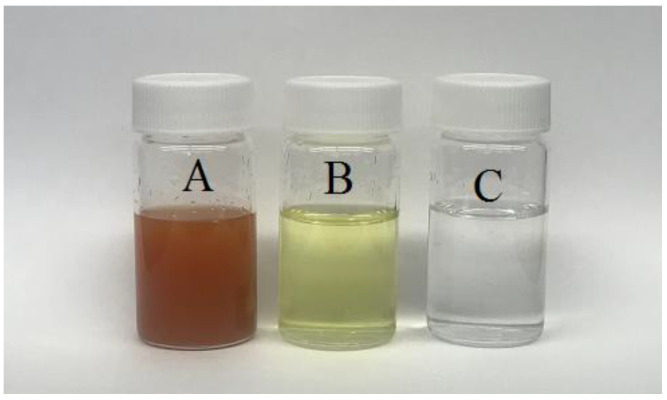
Images of the produced water sample: (**A**) before filtration, (**B**) after filtration, and (**C**) with HEDP after filtration.

The water sample was first filtered with Whatman-41 filter paper to remove the large solid particles. The antiscalant HEDP (50 mg/L) was added to the filtered water prior to the DCMD experiment. The results of the inductively coupled plasma-mass spectrometry (ICP-MS) analysis of the filtered produced water is shown in Table 1. It is clear from Table 1 that the filtration process removed a significant amount of salts that were present as suspension. Figure 1 shows the produced water sample before, after filtration, and with HEDP antiscalant. As can be seen, the addition of HEDP to the filtered solution also changed the color of the solution, mainly due to the sequestration of iron.

### 2.4. CNIM Fabrication

Effective dispersal of CNTs and immobilization on the membrane surface was an essential step in CNIM fabrication. The CNIM was prepared using a polytetrafluoroethylene (PTFE) laminate supported on polypropylene composite membrane (Advantec, 0.2 μm poresize, 74% porosity, Dublin, CA, USA). The dispersion of the CNTs was carried out as follows: 1.5 mg of CNTs were dispersed in a solution containing 8 g of acetone and sonicated for four hours. A total of 0.2 mg of polyvinylidene di fluoride (PVDF), which acted as a binder during immobilization of the CNTs was dissolved in 2 g of acetone and mixed with CNTs dispersion as mentioned in our previous papers [32]. PVDF-nanotube dispersion was thereafter applied uniformly with a dropper over the membrane held on a flat surface to form the CNIM. The wet CNIM was kept under the hood for overnight drying. The CNIM was characterized using scanning electron microscopy (SEM) Leo 1530 VP, Carl Zeiss SMT AG Company, Oberkochen, Germany.

### 2.5. Experimental Procedure

**Figure 2:**
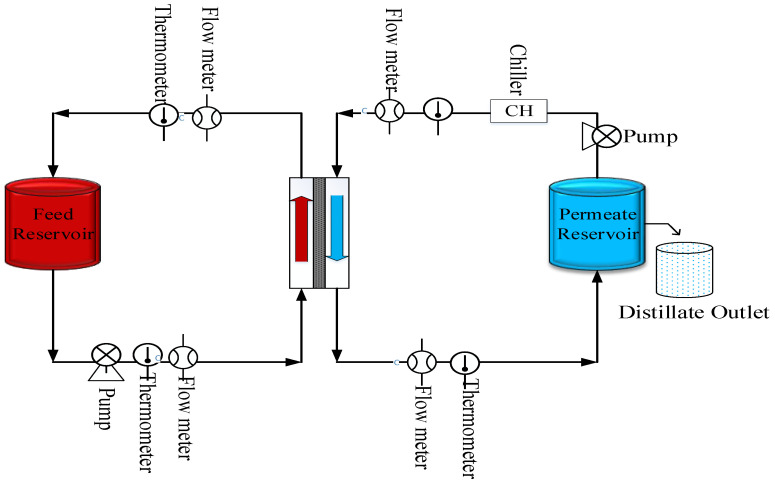
Schematic representation of the experimental setup.

MD experiments were conducted in the DCMD configuration. Figure 2 shows the schematic diagram of the MD system used in the laboratory. The membrane module used for DCMD was a cylindrical module utilizing a flat membrane with a gasket diameter of 3.9 cm and an effective membrane area of 11.94 cm^2^. The preheated hot produced water was passed through a heat exchanger, which was used to maintain the desired temperature throughout the experiment. The hot feed was recycled to the feed tank and permeate was collected in the distillate tank. DI water was used as cold distillate. Both hot and cold sides were circulated through the module using peristaltic pumps (Cole Parmer, model 7518-60, Vernon Hills, IL, USA). Inlet and outlet temperatures of the feed and distillate were monitored continuously throughout the experiment. Viton and PFA tubing and connectors (Cole Parmer) were used to make connections in the experimental set up. The ionic strength of the original feed solution and permeate were measured using an electrode conductivity meter (Jenway4310). The hydrophobic/hydrophilic characteristic of the membrane was evaluated using contact angle measurements. Distilled water and filtered produced water samples were used for this experiment. A micro syringe (Hamilton 0−100 μL) was used to create 2 μL droplets (as described in our previous paper [50].

### 2.6. DCMD Performance Using CNIM and PTFE Membrane

The MD performances of the PTFE and CNIM was studied as a function of time, temperature, and feed flow rate. The water vapor flux, *J_w_* was measured as:

(1) Jw=Wpt×A

where *W_p_* is the mass of permeated water in time *t* through surface area *A*.

To compare the fouling on both membranes, the flux measured over time and the normalized flux decline, *FD_n_* was measured as:

(2) FDn(%)=(1−JfJ0)×100

where, *J_f_* and *J*_0_ are the final permeate flux and initial flux, respectively.

## 3. Results and Discussions

### 3.1. Membrane Characterization

**Figure 3:**
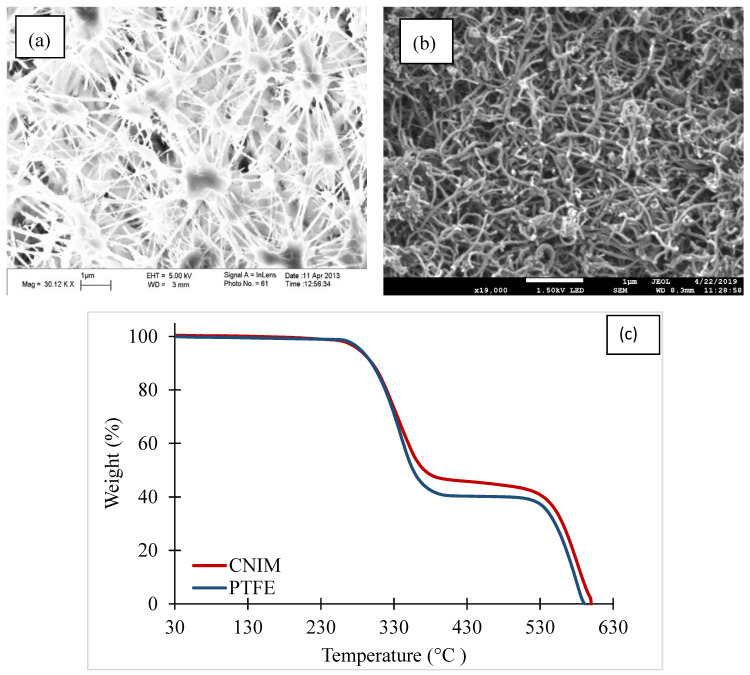
(**a**) SEM image of PTFE membrane, (**b**) CNIM, and (**c**) The TGA curves of PTFE membrane and CNIM.

The SEM images of the CNIM and the PTFE membranes are shown in Figure 3a,b. The SEM images show the porous structure of the pristine PTFE membrane and presence of CNTs on the CNIM surface. The distribution of CNTs is relatively uniform over the entire membrane surface. AFM images from our previous studies have shown that the incorporation of CNTs on the membrane surface also increases the surface roughness [51]. The gas permeation test of the membranes demonstrated no significant change in the effective surface porosity over the effective pore length of the membranes, as only a small quantity of CNTs was immobilized on the membrane surface [50]. AFM images and the gas permeation test data are not presented here for brevity. The thermogravimetric analysis (TGA) curves of PTFE and CNIM membranes are shown in Figure 3c. It is clear from the figure that the membranes are quite stable within the experimental temperature ranges. The initial weight loss of the membrane, in the range of 250 °C to 350 °C, is mainly due to the degradation of the support polypropylene (PP) layer followed by the degradation of the active PTFE layer at 530 °C. Since, the support layer thickness (or amount) was higher than the active PTFE layer, the first degradation was observed to be higher. The TGA curve for CNIM shifted slightly upward, which showed the enhanced thermal stability of the CNIM due to the presence of CNTs [52].

**Figure 4:**
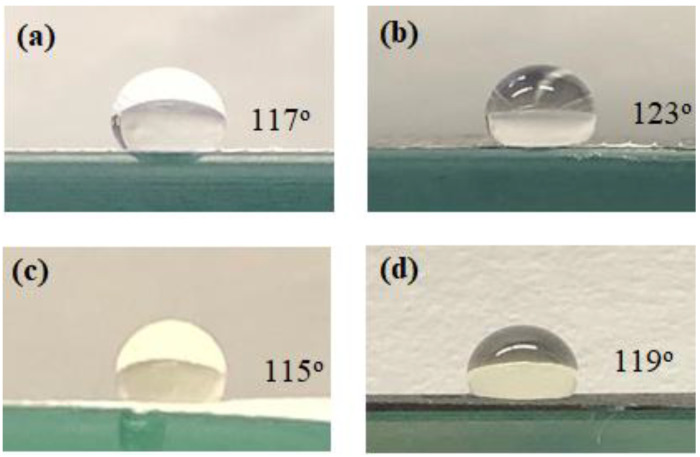
Photograph of pure water on (**a**) unmodified PTFE (**b**) CNIM and produced water solution on (**c**) unmodified PTFE (**d**) CNIM.

The photographs of the contact angles of the PTFE and CNIM for pure water and the salt solution are shown in Figure 4. A droplet size of 4 mm was used to measure the contact angles. The presence of CNTs dramatically altered the contact angle. With 100% water in the feed (Figure 4a,b), the contact angle for CNIM was higher (123°) than the PTFE membrane (117°). In the presence of different foulants, such as dispersed oil, organic acids, polycyclic aromatic hydrocarbons (PAHs), and phenols, the contact angle decreased slightly (Figure 4c,d). The liquid entry pressure (LEP) was measured using a method described before [40]. The LEP of the pure water solution was found to be 75 and 71 psig, which changed to 69 and 65 psig with the produced water solution for unmodified PTFE and CNIM, respectively. The lower LEP for CNIM may be due to the presence of organic moieties in the produced water.

### 3.2. Effect of Temperature and Feed Flow Rate on Water Vapor Flux

**Figure 5:**
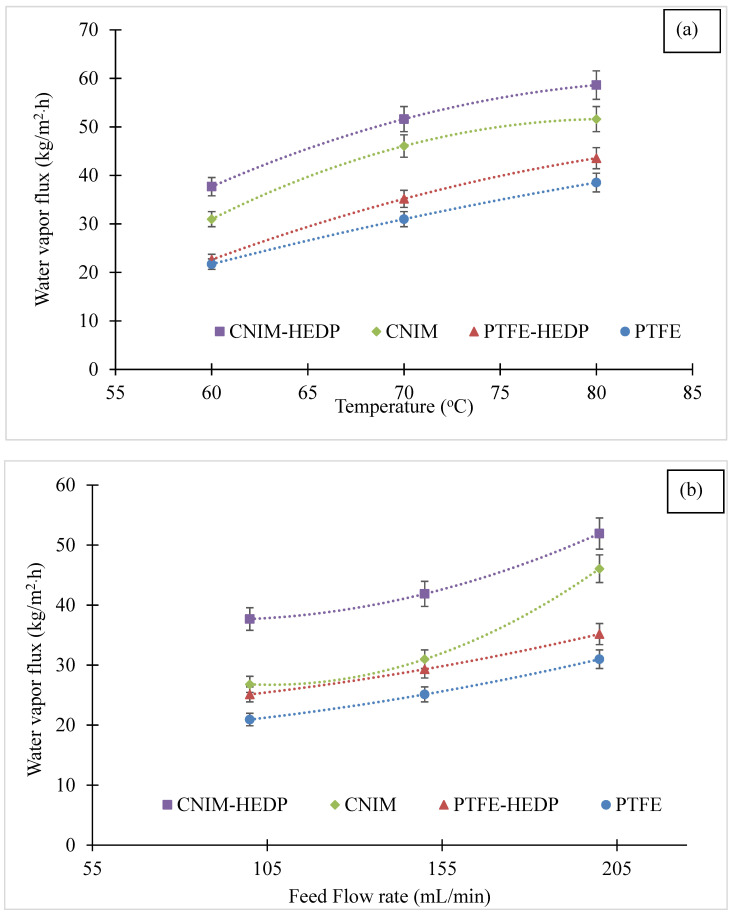
(**a**) Effect of temperature on permeate flux of produced water solution at 200 mL/min flow rate. (**b**) Effect of flow rate on permeate flux of produced water solution at 70 °C temperature and 200 mL/min flow rate.

The effect of temperature on permeate flux for both membranes is illustrated in Figure 5a at a feed flow rate of 200 mL/min and distillate flow rate of 200 mL/min1. It is clear from the figure that the water vapor flux increased with an increase in temperature as higher feed temperature generates a high vapor pressure gradient. The CNIM demonstrates higher flux compared to the PTFE membrane at all temperatures. The flux enhancement in CNIM was mainly due to the fact that the CNTs serves as effective sorbent sites for water vapor transport while repelling the liquid water. This was followed by activated diffusion on the smooth CNTs surfaces, which was in line with our previously reported data [30,34,40,51]. Further, the addition of HEDP on the feed side led to an enhanced MD performance for both membranes. At a temperature of 70 °C, the water vapor flux increased from 30.9 to 35.2 kg/m^2^·h for PTFE and 46.1 to 51.6 kg/m^2^·h with CNIM with the use of HEDP with the same experimental conditions.

The results of increasing feed flow rate at a constant temperature of 70 °C and 200 mL/min permeate flow rate is shown in Figure 5b. It was observed that the permeate flux increased with an increase in feed flow rate for all membranes and the CNIM had higher water vapor flux when compared to PTFE. The increased feed flow rate reduced the fouling by increasing the turbulence, which in turn reduced the boundary layer effect at the membrane-feed solution interface [53]. As can be seen from Figure 5b, at a feed flow rate of 150 mL/min and with the use of HEDP, the water vapor flux increased from 25.1 to 29.3 kg/m^2^·h for PTFE and from 30.9 to 41.9 kg/m^2^·h with CNIM, which was a 17% and 36% improvement, respectively.

### 3.3. Fouling Behavior of Produced Water

**Figure 6:**
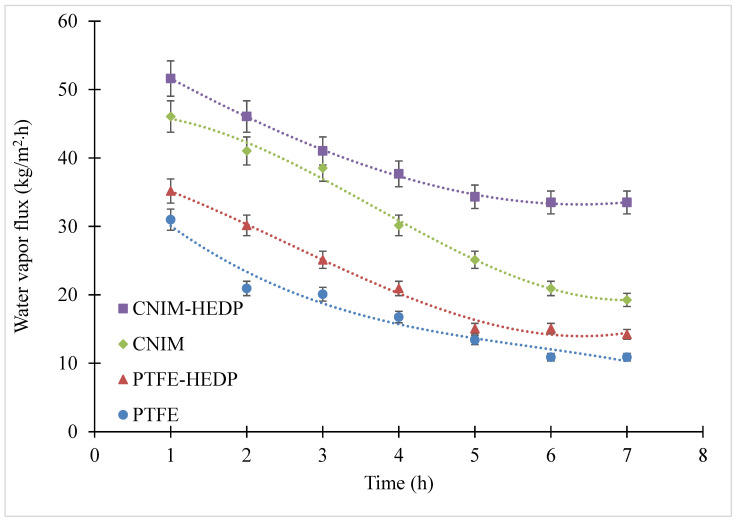
Water vapor flux in PTFE and CNIM membranes for produced water solution with and without using HEDP (antiscalant).

The fouling behavior of the produced water was studied on the PTFE membrane and CNIM using HEDP antiscalant and was characterized by the reduction in the permeated water flux as a function of time. Figure 6 shows that the water vapor flux decreased significantly with time for all membranes as an outcome of scaling. It is clear from the figure that the CNIM exhibited higher antifouling properties in comparison with the PTFE membrane. This may be due to the additional screening effect of CNTs, which reduce pore blocking from salt deposition on membrane pores. For the produced water solution, the flux declined to 10.9 from 30.9 kg/m^2^·h for PTFE and to 19.3 from 46.1 kg/m^2^·h for the CNIM after 7 h of operation. The results show that by using CNIM, the water vapor flux after 7 h was still 77% higher than that of the PTFE membrane. The use of HEDP in the feed solution further improved the antifouling behavior of both membranes and the water vapor flux after 7 h of operation was 35.2 kg/m^2^·h and 51.6 kg/m^2^·h for the PTFE membrane and CNIM respectively, which were 32.4% and 74% higher compared to the system without HEDP. This may be due to the fact that the antiscalant delayed the clustering process and prevented the precipitation of salt on the membrane surface [54].

The normalize flux decline (FDn) for all membranes after 7 h of operation with produced water are shown in Table 2. It is clear from the table that the reduction in flux was lower in CNIM compared to the PTFE membrane, which indicates a lower fouling tendency. The use of HEDP further improved the antifouling property of both membranes. Under similar conditions, the CNIM with HEDP exhibited an improvement of 41% in FDn compared to the pristine PTFE membrane.

### 3.4. Deposition of Foulants on the Membrane Surface

The deposition of the foulants on the membrane surface was evaluated by measuring the membrane weight before and after the experiment. The weight measurements were done with precision by drying the membrane overnight in the oven at 70 °C to avoid any loss of deposited foulants from the surface.

From Table 3 it is clear that less salt was deposited on the membrane surface for CNIM than for the pristine PTFE membrane for all cases. The lower salt deposition on CNIM may be attributable to the screening effect of CNTs. The table also demonstrates the advantage of using antiscalant in reducing salt deposition on the membrane surface. The CNIM along with HEDP in the feed, successfully lowered the salt deposition on the membrane surface, which is a major concern in treating feed containing higher amount of foulants, such as in produced water.

**Figure 7:**
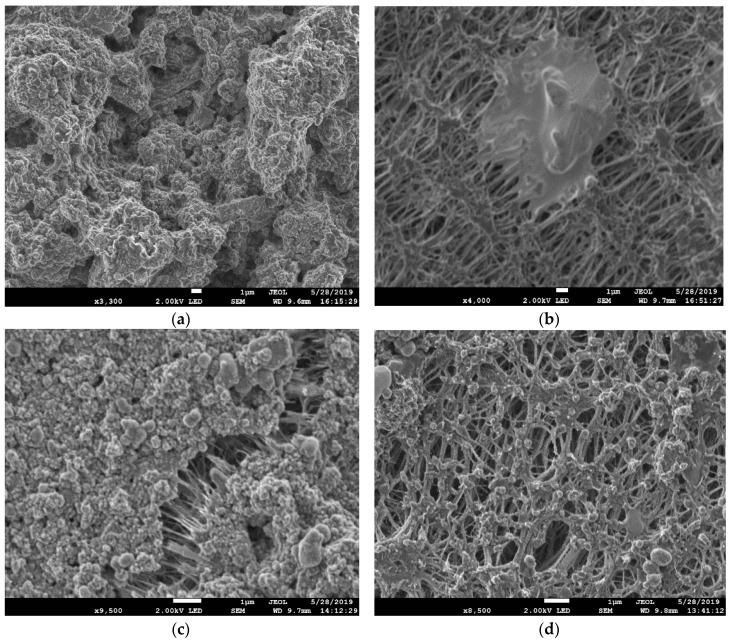
SEM image of foulant deposition on (**a**) PTFE, (**b**) PTFE-HEDP, (**c**) CNIM and (**d**) CNIM-HEDP.

SEM images of the deposition of various salt crystals on different membrane surfaces with and without using HEDP antiscalant are shown in Figure 7. The SEM images clearly show the variation in the foulants’ morphology and amounts deposited on the membrane surfaces, with and without using HEDP for both membranes. The images also reveal that the use of HEDP significantly reduced the fouling layer on the membrane surface. The antiscalant interacts with the foulants and with the membrane surface to break down the crystals (foulants) and reduce the fouling as is shown in Figure 7b,d.

### 3.5. Membrane Regeneration

The regenerability of the fouled PTFE membrane and CNIM treating the produced water was studied with and without HEDP (antiscalant). Here, the MD experiments were run continuously for 6 h followed by washing the fouled membrane for 30 min with DI water at 70 °C, then this cycle was repeated and the flux after 24 h is shown in Table 4.

Table 4 shows the regenerability of the membranes with produced water, with and without HEDP. It is clear from the table that the CNIM was able to attain around 91% of its initial water vapor flux, which clearly indicates significant removal of the deposited salts from the membrane surface and pores. In contrast, the PTFE membrane only reached up to 81% of its original value, which clearly demonstrates the superiority of CNIM in terms of membrane regeneration. The use of HEDF further helped regeneration of both membranes, which showed around 95% recovery of the initial flux.

### 3.6. Mass Transfer Coefficient

The overall, mass transfer coefficient can be described as:

(3) $$Jw\;=\;k\;\left(P_{f}\;-\;P_{p}\right)$$

or,

(4) $$k\;=\;Jw/\left(P_{f}-P_{p}\right)$$

where, *Jw* is the water vapor flux, *k* is the mass transfer coefficient, and *P_f_* and *P_p_* are the partial vapor pressure of the average feed and permeate temperatures. The mass transfer coefficients were found to be higher for the CNIM membrane as compared to the PTFE membrane.

Table 5a summarizes the change in mass transfer coefficients of PTFE membrane and CNIM with varying feed flow rates at 70 °C. Both membranes exhibited an increased mass transfer coefficient with an increase in the feed flow rate. The diffusion of the water vapor through the boundary layers mainly controls the overall mass transfer rate of the process. At a higher feed flow rate, the turbulence increased, which led to the reduction in the boundary layer resistance and significantly increased the mass transfer coefficients. Of these two membranes, CNIM exhibited higher mass transfer coefficients compared to the PTFE membrane.

Table 5b shows the mass transfer coefficients at different temperatures. It is evident from the table that the CNIM showed higher mass transfer coefficients compared to the pristine PTFE membrane in all cases. Rapid sorption/desorption on CNTs surfaces along with activated diffusion led to an increase in the overall water vapor transport. The mass transfer coefficients were found to decrease with an increase in temperature for both membranes. This may be due to the fact that with the increase in feed temperature, the concentration polarization at the membrane surface was expected to increase, resulting in higher resistance for mass transfer on the feed side of membrane. Further, at higher temperatures, the membrane fouling rate was also observed to increase at higher temperature [55,56].

## 4. Membrane Stability

The quality of the permeate side water was carefully investigated to monitor the stability of modified membrane and salt breakthrough. The stability of CNIM was tested for 60 days. The permeated water was monitored throughout the experiment to ensure the quality of water by measuring the conductivity of the permeate side water and using Raman spectroscopy [57]. The permeated water sample did not show any presence of salts or CNTs after a long period of operation.

## 5. Proposed Mechanism

**Figure 8:**
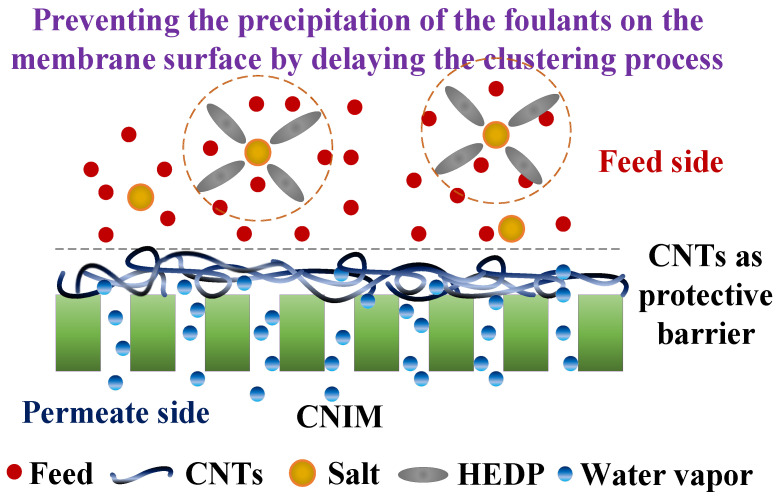
Schematic of the proposed mechanism.

The proposed mechanism for enhanced antifouling behavior of CNIM in presence of HEDP antiscalant is shown in Figure 8. HEDP is known to be one of the most effective threshold inhibitors based on phosphonic acids (or their salts), which prevents the precipitation of the foulants on the membrane surface by delaying the clustering process of charged ions protonuclei [54]. This is important in membrane applications, especially with produced water as it contains large quantity of inorganic salts that deposit on the membrane surface, causing a significant reduction in membrane performance and wetting. Our previous studies with CNTs have demonstrated that CNTs are excellent sorbents that enhance partition coefficient of the solutes leading to higher flux in membranes [31,34,51,58]. Further, the presence of CNTs could act as an additional screen that also prevents the salt deposition on the membrane pores.

## 6. Conclusions

The CNIM was successfully employed in treating the produced water. The MD performance of CNIM was compared with pristine PTFE membrane. The addition of HEDP antiscalant in the produced water feed solution, further helped to reduce fouling and prevented the deposition of foulants on the membrane surface. The CNIM exhibited a lower flux decline and the regenerability of the CNIM was also found to be superior to that of the pristine PTFE membrane. In summary, treating the produced water solution using HEDP antiscalant on CNIM in MD was found to be highly effective in reducing the fouling behavior, which in turn led to an enhancement in water vapor permeation through the membrane.

## Figures and Tables

**Table 1:** Analysis of the original produced water sample and after filtration.

Parameters/Dissolved Solids (mg/L)	Produced Water	After Filtration
Calcium	119,500	1455
Magnesium	12,590	52
Barium	856	289
Iron	90	1.4
Copper	<1.0	0.014
Zinc	2.4	0.025
Sodium	71,820	154
Potassium	1780	13
Chloride	118	-
Sulfate	130	-
Nitrate	<100	-
Ortho−Phosphate	<500	-
Silica	41	-

**Table 2:** Normalized flux decline (FDn) for the produced water solution.

Solution	FDn (%) of Produced Water Solution			
	PTFE	CNIM	PTFE-HEDP	CNIM-HEDP
Produced Water	64.8	58.2	59.5	35.1

**Table 3:** Deposition of foulants on the membrane surface after 7 h of operation at 70 °C.

Solution	Amount of Salt Deposited on the Membrane Surface (mg)		% Weight Decrease
	PTFE	PTFE-HEDP	
Produced Water	15.76	4.68	70.3
	CNIM	CNIM-HEDP	
	1.02	0.79	22.5

**Table 4:** Membrane regeneration data.

Membrane	Initial Flux (kg/m^2^·h)	Flux after 24 h (kg/m^2^·h)	Flux Regenerated (%)
PTFE	30.9	25.1	81.1
PTFE-HEDP	35.2	33.5	95.2
CNIM	46.1	41.9	90.9
CNIM-HEDP	51.9	49.4	95.2

**Table 5:** (**a**) Effect of varying feed flow rate on mass transfer coefficient at 70 °C. (**b**) Mass transfer coefficient of various membranes as a function of temperature.

**(a)**		
**Mass Transfer Coefficient (kg/m^2^ sec^−1^ Pa) × 10^−7^**		
**Feed Flow Rate (mL/min)**	**PTFE**	**CNIM**
100	1.9	2.6
150	2.4	3.0
200	3.7	4.4
**(b)**		
**Mass Transfer Coefficient (kg/m^2^ sec^−1^ Pa) × 10^−7^**		
**Temperature (°C)**	**PTFE**	**CNIM**
60	3.4	4.9
70	3.0	4.4
80	2.4	3.1
